# Laparoscopic and endoscopic cooperative surgery for gastric cancer mimicking a submucosal tumor

**DOI:** 10.1186/s40792-020-00855-4

**Published:** 2020-05-11

**Authors:** Hiroki Ozawa, Hirofumi Kawakubo, Satoru Matsuda, Shuhei Mayanagi, Tomoyuki Irino, Kazumasa Fukuda, Rieko Nakamura, Norihito Wada, Yuko Kitagawa

**Affiliations:** Department of Surgery, Keio University School of Medicine, 35 Shinanomachi, Shinjuku-ku, Tokyo, 160-8582 Japan

**Keywords:** LECS, Gastric cancer, Gastric cancer simulating SMT

## Abstract

**Abstract:**

**Background:**

Gastric cancer that mimics a submucosal tumor (SMT) is infrequently encountered in routine clinical settings, and histopathological analysis is often negative for malignant cells. In such cases, excisional biopsy of the entire tumor may be necessary to make a definitive pathological diagnosis, and laparoscopic and endoscopic cooperative surgery (LECS) is a viable method of excisional biopsy.

**Case presentation:**

An 80-year-old male patient diagnosed with stomach wall irregularities at routine medical check-up was referred to our facility, and consequent endoscopic examination detected a 20-mm protruded lesion in the greater curvature at the middle third of the stomach. Endoscopic ultrasound (EUS) showed a thick, low echoic lesion with an irregular margin in the second layer of the gastric wall. Further, a nodular part of the lesion had infiltrated into the submucosa, with an appearance similar to that of linitis plastica of the stomach. The lesion was highly suspected to be a gastric carcinoma with submucosal invasion. However, mucosal-incision-assisted biopsy revealed no malignant cells. Computed tomography (CT) identified no metastatic lymph nodes. Therefore, an excisional biopsy using LECS was performed, and to avoid peritoneal dissemination, we used a modified version, namely, combination of laparoscopic and endoscopic approaches to neoplasia with non-exposure technique (CLEAN-NET). The procedure ended without any complications, and post-operative course was uneventful. As histopathology returned a diagnosis of adenocarcinoma pT4a, we performed radical gastrectomy and D2 lymphadenectomy. Post-operative course was unremarkable and the patient underwent follow-up examinations without adjuvant chemotherapy because of old age.

**Conclusions:**

Local resection using LECS for gastric tumors with a high suspicion of malignancy is useful and feasible. LECS could be used in similar cases.

## Background

Laparoscopic and endoscopic cooperative surgery (LECS) is a procedure that combines laparoscopic gastric resection and endoscopic submucosal dissection (ESD) with appropriate but minimal surgical resection. LECS is routinely performed for local resection of gastric submucosal tumors (SMTs), including gastrointestinal stromal tumors (GIST) [1]. In intraluminal tumors, it is difficult to recognize tumor location and determine resection margin during conventional laparoscopic surgery alone. However, LECS could help in determining accurate cutting margins using a combination of laparoscopic and endoscopic views, and therefore, this procedure can be expected to preserve gastric function without excessive resection. The indications for LECS use have been expanding, and modified LECS procedures have been developed for certain kinds of gastric cancer. Further, LECS in combination with sentinel node navigation surgery (SNNS) has been developed, and its efficacy was tested in a clinical trial [2]. Here, we describe the successful use of LECS to diagnose and excise a SMT-like tumor. As the lesion was histopathologically determined to be advanced gastric cancer, radical gastrectomy was also subsequently performed.

## Case presentation

**Fig. 1:**
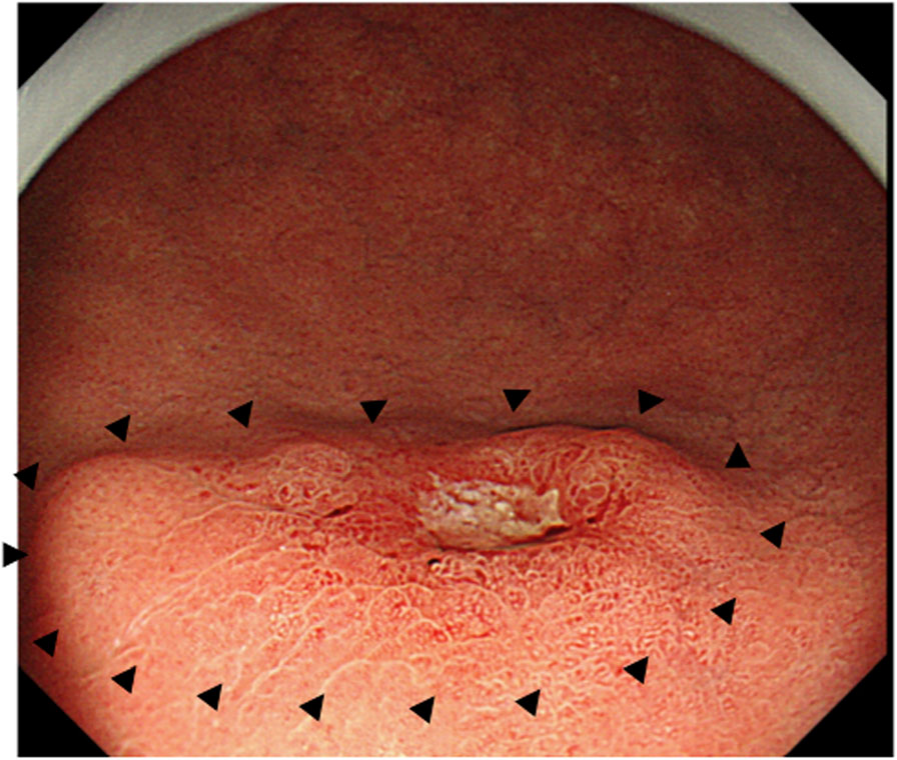
Endoscopic examination detected a 20-mm-sized protruded lesion in the greater curvature at the middle third of the stomach. There was a depression with ulcer on the top of the tumor. The black triangles indicate the expected border

**Fig. 2:**
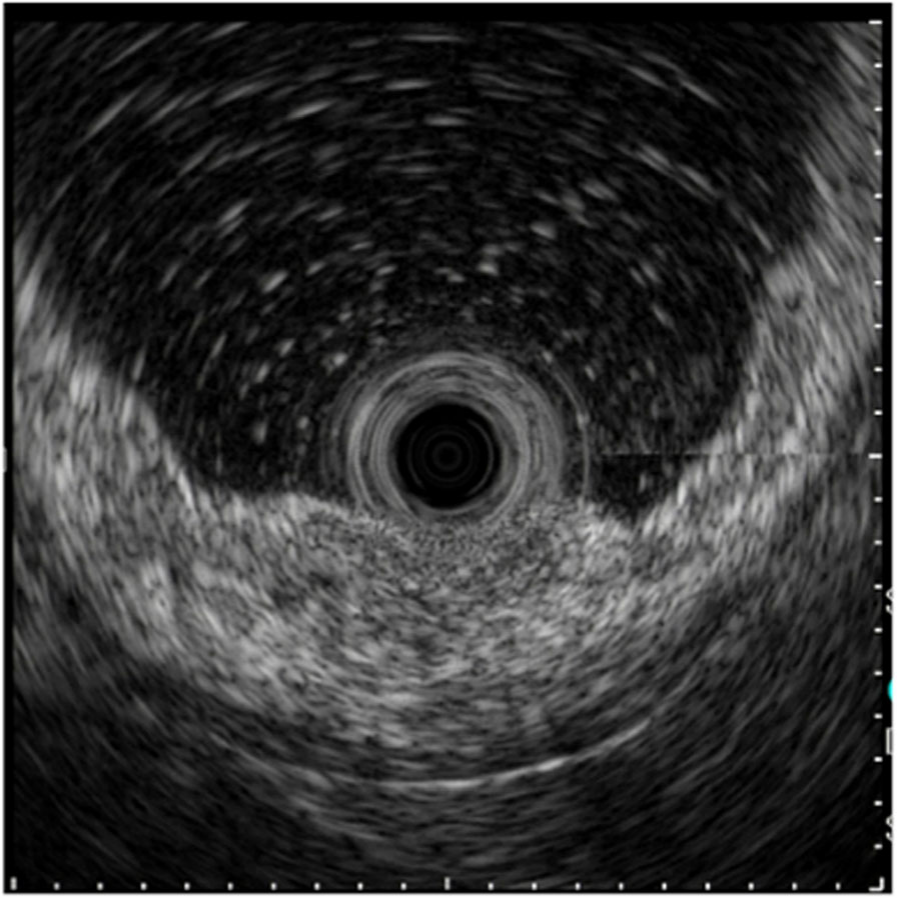
EUS detected a thick, low echoic lesion in the second layer of the gastric wall, and part of the tumor had infiltrated into the submucosa with the nodule

An 80-year-old male patient who was found to have stomach wall irregularities during routine medical check-up was referred to Keio University Hospital. Endoscopic examination detected a 20-mm protruding lesion in the greater curvature at the middle third of the stomach (Fig. 1). The tumor was almost covered by normal mucosa, and the top of the lesion was partly depressed. Narrow-band imaging (NBI) showed the vascular surface pattern to be irregular. Further, EUS showed a thick, low echoic lesion in the second layer of the gastric wall, and the nodular part of the lesion had infiltrated into the submucosa (Fig. 2). On the basis of the above findings, we suspected the tumor to be gastric cancer mimicking SMT, similar to linitis plastica of the stomach with SM invasion or a lymphoproliferative disorder such as a malignant lymphoma. However, the endoscopic mucosal, boring, and mucosal-incision-assisted biopsies revealed only a small number of atypical epithelial cells and no malignant cells. Additionally, CT and fluorodeoxyglucose positron emission tomography (PET) showed no lymph node metastasis. We diagnosed the tumor to be a gastric submucosal tumor with a high suspicion of malignancy based on the qualitative diagnosis. Therefore, an excisional biopsy was planned. Using endoscopic diagnosis as the reference, ESD was not selected because a conventional radical gastrectomy would have been highly invasive. Instead, we planned a partial gastrectomy using LECS for tumor resection.

To avoid peritoneal dissemination, we chose the modified CLEAN-NET procedure [3]. Since the EUS revealed that the tumor was unlikely to spread laterally, we planned to excise the raised part only. The cutting line was decided to be 4 mm outside of the expected border.

**Fig. 3:**
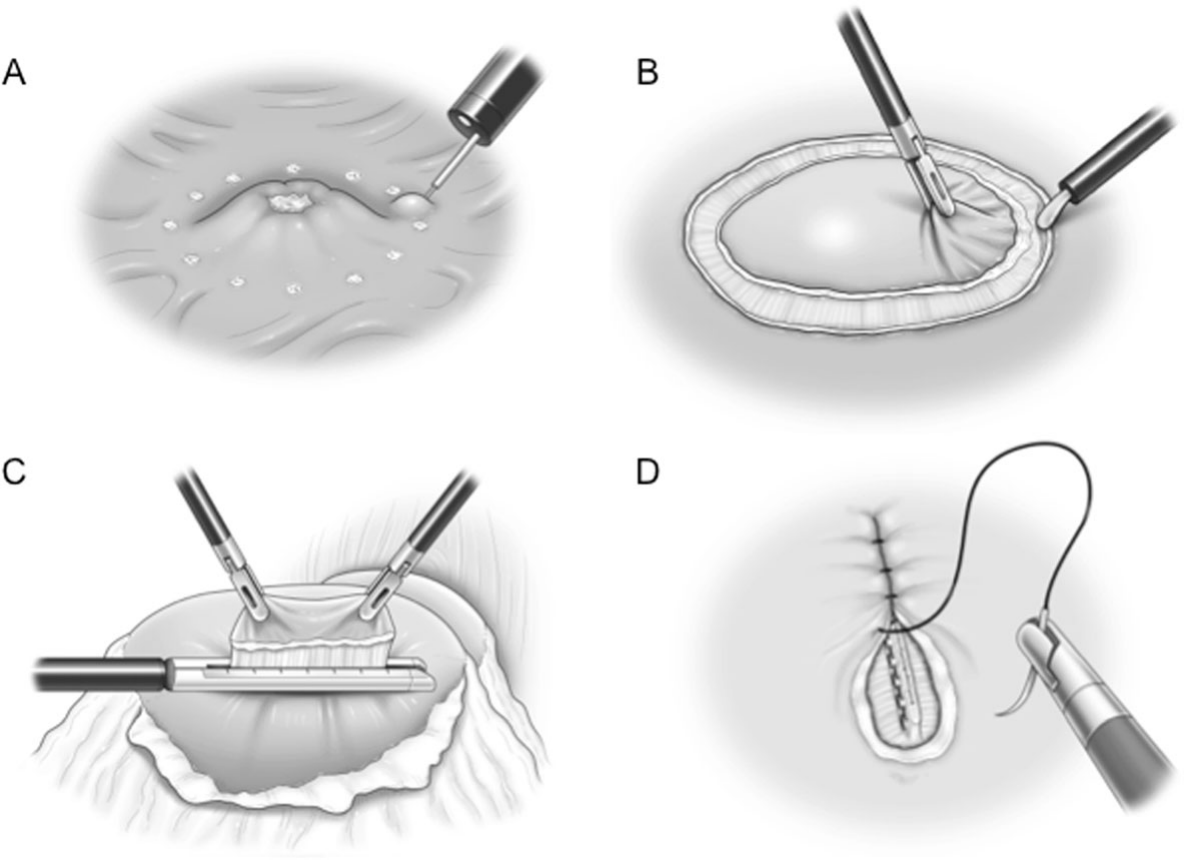
Intraoperative findings. **a** Mucosal marking around the tumor and injection of sodium hyaluronate solution with indigo carmine around the tumor under endoscope guidance. **b** A circumferential seromuscular incision around the tumor. **c** Dissecting a full-layer specimen using a laparoscopic-stapling device. **d** Linear suturing of the seromuscular layers

**Fig. 4:**
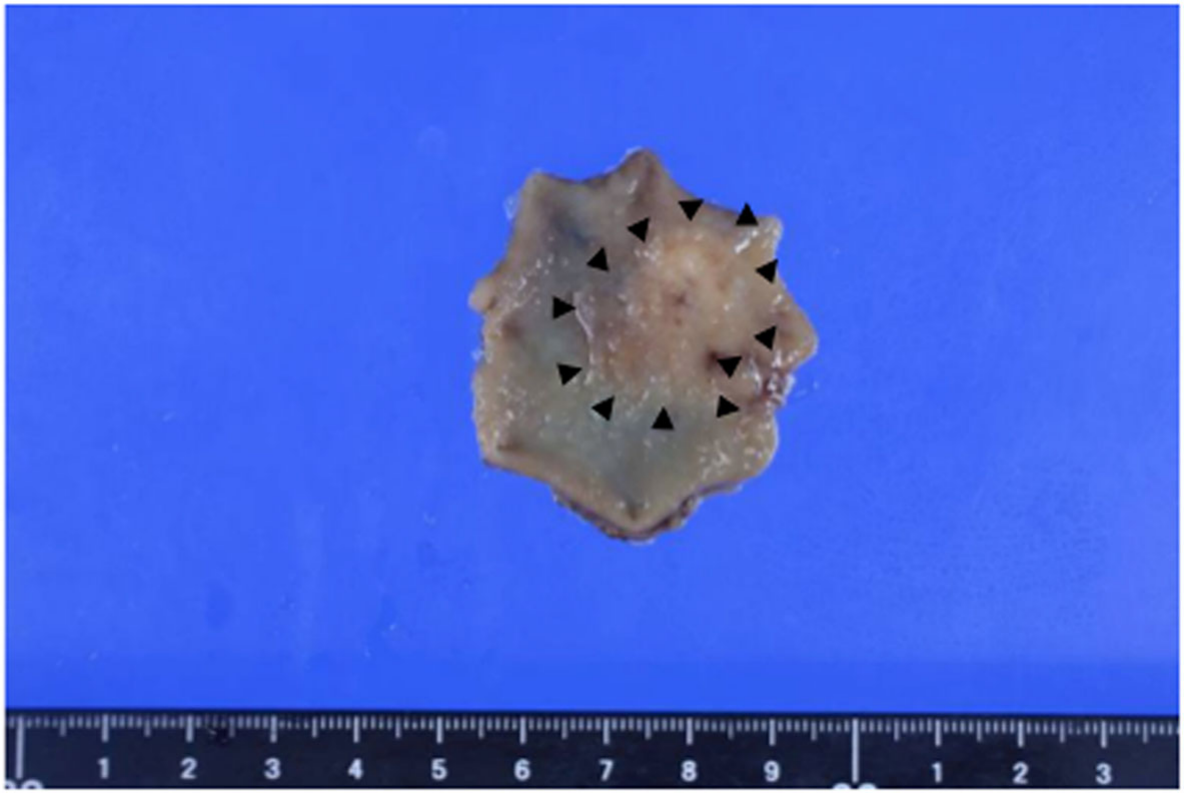
Pathological specimen. The tumor was resected with negative lateral margins. Almost all of the resected mucosa appeared flat and smooth. The black triangles indicate the actual border of the cancerous region

The technical details of this surgery involve (1) detecting the lesion and performing an endoscopic submucosal injection, (2) performing seromuscular dissection around the tumor under laparoscopic view, (3) cutting the mucosal layer using a mechanical stapler (Fig. 3), (4) and suturing the seromuscular layer.

The procedure duration was 51 min, blood loss was 0 mL, and there is no perforation of the gastric wall. The patient’s postoperative course was uneventful. Histopathological analysis of the specimen identified the tumor to be a moderately differentiated adenocarcinoma, pT4a(SE), Ly1a, V0, negative lateral margins (Fig. 4). On the basis of these results, the tumor was classified as pT4a cN0M0 cStage IIB (according to TNM classification, 8th ed.), and an additional radical gastrectomy was planned.

A robotic-assisted distal gastrectomy with D2 lymphadenectomy was performed 6 weeks after the first procedure. The duration of the procedure was 338 min, and the blood loss was 50 mL. Adhesions from the previous procedure were not severe, and the postoperative course was uneventful. At the time of the final pathological diagnosis, there was no metastasis in the regional lymph nodes and no residual carcinoma in the resected stomach. The patient refused adjuvant chemotherapy of S-1 because of his old age and underwent follow-up examinations every 6 months.

## Discussion

LECS is a procedure that combines laparoscopic gastric resection with endoscopic submucosal dissection for local excision of gastric tumors with minimal surgical resection margins [4]. This simultaneous intraluminal approach with endoscopy allows surgeons to optimize resection area, and thus, LECS can be expected to preserve gastric function without excessive resection [5]. Additionally, LECS is a minimally invasive procedure that allows oncologically precise resection. Initially, gastric fluid leakage and dissemination of the tumor cells were problems that prevented the use of LECS as a treatment modality for gastric cancer [4]. However, these problems have largely been overcome by the development of modified LECS procedures such as the inverted LECS [6], closed LECS [7], CLEAN-NET [3], and non-exposed endoscopic wall-invasion surgery (NEWS) [8]. There are several reports on the use of LECS for gastric cancer [3, 4, 6, 8–10] (Table 1). In our institution, we primarily choose the modified CLEAN-NET procedure for extra-luminal or intramural gastric SMTs. Unlike the original method, we resected the mucosal layer using a mechanical stapler, and then manually sutured the seromuscular layer to further reduce gastric deformation and prevent hardening of the suture line site.

**Table 1:** Clinical outcomes of LECS for gastric cancer

Author	Publish year	Patient number	Age	Gender (Male/Female)	Procedures	cT Diagnosis	Size (mm)	Conversion to Gastrectomy	Positive surgical margin	Mortality	Recurrence	Follow up period (Month)
Nunobe et al [6]	2012	1	70	0/1	I-LECS	pT1a	60	0	0	0	-	-
Inoue et al [3]	2012	16	66.2^a^	-	CLEAN-NET	pT1a-T1b	-	-	-	0	0	-
Goto et al [8]	2015	1	55	0/1	NEWS	pT1a	20	0	0	0	0	-
Aoki et al [9]	2018	4	78.8^a^	4/0	C-LECS	pT1a	14.5^a^	0	0	0	0	33.5^a^
		3	79^a^	3/0	I-LECS	pT1a	11.7^a^	0	0	0	0	12.6^a^
Yoshida [10]	2018	1	54	1/0	I-LECS	pT1a	7	0	0	0	0	-
Takechi [11]	2018	1	68	0/1	I-LECS	pT2	50	0	0	0	0	6
Our case	2019	1	80	1/0	CLEAN-NET	pT4a	20	0	0	0	0	-

^a^MEAN, *C-LECS* Classical LECS, *I-LECS* Inverted LECS, *NEWS* Non-exposed Endoscopic Wall-inversion Surgery, *CLEAN-NET* Combination of laparoscopic and endoscopic approaches to neoplasia with non-exposure technique

Additionally, the LECS concept is beginning to be used for tumor excision in other organs such as the duodenum, the colon, and the rectum [4].

The indications for LECS are currently limited, and the clinical settings in gastric cancer that are amenable to LECS are as follows.

The first setting corresponds to lesions that are normally managed by ESD but are technically challenging to resect via ESD, for example, because of the presence of ulcers or tumor location and size, for instance, if the tumor size is more than 30 mm in diameter or the tumor is located at the fornix [6]. Aoki et al. reported performing LECS (classical LECS and inverted LECS) in seven cases of intramucosal gastric carcinoma with adjacent ulcer scars [9], and Yoshida et al. described LECS for gastric cancer with severe fibrosis. In the latter study, initial attempts at ESD failed because of gastric perforation, and at 2 months after ESD, local resection using LECS had to be performed because of severe fibrosis that prevented ESD [10]. As for tumor location, ESD for gastric tumors that are located in the fornix or the esophagogastric junction is considered technically demanding. By contrast, LECS has wider indications with respect to tumor location. Although tumors larger than 30 mm cannot usually be resected by ESD, Nunobe et al. reported using LECS for a 60-mm gastric tumor located in the fornix [6].

The second indication for LECS is palliative surgery in high-risk cases. In elderly or high-risk patients with heart failure or renal failure, palliative gastrectomy is recommended to prevent complications due to bleeding or perforation. Takechi et al. described inverted-LECS as a palliative treatment for advanced gastric cancer in high-risk patients with liver cirrhosis (Child-Pugh score B), aortic stenosis, and coronary stenosis [11].

The third scenario entails combining LECS with sentinel node navigation surgery (SNNS). Takeuchi et al. demonstrated the feasibility and accuracy of sentinel node biopsy in early gastric cancer [2]. The sentinel node is defined as the first lymph node that receives lymphatic drainage from the primary tumor site, and SNNS for gastric cancer has been validated in a prospective multicenter trial. When a modified LECS procedure and SNNS were combined, an extremely minimally invasive procedure that is adequate for radical oncological resection of early gastric cancer is achieved.

The fourth indication is a tumor requiring diagnostic resection because of high suspicion of malignancy, e.g., gastric cancer mimicking SMT, similar to the case described here. Gastric cancers resembling SMT are very rare because they are epithelial neoplasms that originate in the lamina propria and are usually identified based on mucosal changes. Nonetheless, these tumors sometimes macroscopically mimic SMTs [12] and reportedly account for 0.1–0.63% of all resected gastric cancers in Japan [13]. It may sometimes be difficult to diagnose gastric SMTs using endoscopic biopsy alone even though EUS can shed light on tumor characteristics. However, it is impossible to make a pathological diagnosis of SMT without a tumor sample obtained via fine-needle aspiration (FNA), which is considered a relatively reliable method. Further, the sensitivity, specificity, negative predictive value, and positive predictive value of EUS for the diagnosis of SMT are relatively high (64%, 80%, 78%, and 87%, respectively) [14]. In clinical settings, EUS has become a reliable method for predicting invasion depth in early gastric cancer with accuracy rates of 75% for mucosal and 62% for submucosal cancers. However, Okada et al. reported that findings of ulceration and large tumors are associated with incorrect diagnosis of tumor invasion depth by EUS [15]. Thus, endoscopic ultrasound-guided fine-needle aspiration (EUS-FNA) may be required. In the case described here, a mucosal-incision-assisted biopsy was performed, but no malignant cells were found. Further, because the mucosal-incision-assisted biopsy would have almost certainly removed suitable tissue, we recommended a laparoscopic total excisional biopsy with local resection using a modified LECS procedure. This procedure was chosen because of advantages such as adaptability to a similar tumor, minimal invasiveness, and safety. It is important to note here that all possible differential diagnoses, such as malignant lymphoma or carcinoid and other tumors, should be explored while deciding on treatment strategy. If the tumor is diagnosed as benign, it can be resected using minimally invasive procedures. By contrast, peritoneal dissemination is a significant problem during local resection for gastric cancer, but it can be adequately managed using modified LECS procedures such as the inverted LECS, the closed LECS, CLEAN-NET, and NEWS. Thus, LECS is a safe, feasible, and reasonable strategy for resecting gastric tumors with high suspicion of malignancy. To the best of our knowledge, this is the first description of LECS being used for total excisional biopsy for a suspected malignant gastric tumor and radical gastrectomy.

## Conclusion

Total excisional biopsy using a modified LECS procedure for a gastric tumor with a high suspicion of malignancy appears to be useful and may represent a possible treatment option for similar tumors.

## Data Availability

All data generated or analyzed during this study are included in this published article.

## Abbreviations

SMT: Submucosal tumor
LECS: Laparoscopic and endoscopic cooperative surgery
EUS: Endoscopic ultrasound
CT: Computed tomography
CLEAN-NET: Combination of laparoscopic and endoscopic approaches to neoplasia with non-exposure technique
ESD: Endoscopic submucosal dissection
GIST: Gastrointestinal stromal tumors
SNNS: Sentinel node navigation surgery
NBI: Narrow-band imaging
PET: Positron emission tomography
NEWS: Non-exposed endoscopic wall-invasion surgery
FNA: Fine-needle aspiration
